# Hybrid Teamwork: What We Know and Where We Can Go From Here

**DOI:** 10.1177/10464964241279078

**Published:** 2024-09-09

**Authors:** Lisa Handke, Aliza Aldana, Patrícia L. Costa, Thomas A. O’Neill

**Affiliations:** 1 Friedrich-Alexander-Universitat Erlangen-Nurnberg, Nuremberg, Germany; 2 University of Calgary, AB, Canada; 3 ISCTE-Instituto Universitario de Lisboa Unidade de Investigacao em Desenvolvimento Empresarial, Portugal

**Keywords:** virtual teams, hybrid work, technology use

## Abstract

Hybrid teamwork, which describes any combination of one’s work time spent across organizational and other (typically domestic) work settings, has become a critical aspect of modern work environments. However, despite the rising prevalence and technological support for hybrid teamwork, there is limited understanding of its impact at the team level. Although we still lack research that addresses the dynamic geographic configurations inherent to hybrid teamwork, we believe that much of the extant literature on virtual teamwork can inform our understanding and guide future research. Accordingly, this paper aims to advance knowledge on hybrid teamwork by defining its unique characteristics and critically reviewing three broad classes of theory from the virtual teams literature and their implications for understanding hybrid teamwork. Based on both contributions and limitations of these three theory classes, we conclude this paper by mapping out pressing questions to guide future research.

Hybrid teamwork, which has emerged as an omnibus term for teams with members who utilize a degree of both remote and on-site working arrangements (e.g., Bell et al., 2023; Mitchell & Brewer, 2022), has become increasingly prevalent in today’s work environment. Remote work opportunities have become a clear workplace expectation rather than a perk, with the majority of employees wanting to work remotely for 2 to 3 days a week (e.g., Aksoy et al., 2023; Buffer, 2023). In the wake of the pandemic, by the beginning of 2023 26% of full-time employees across the globe were working in hybrid work arrangements (Aksoy et al., 2023). Coupled with rapid technological advances to promote working together at a distance, it is evident that hybrid teamwork has become a collaborative practice that is here to stay (Gibson et al., 2023).

At the same time, even though hybrid work itself has received great attention in both the academic and popular press (e.g., Gratton, 2020, 2021; Hilberath et al., 2020), little has been done to address hybrid work at the team level, meaning that we still do not know enough about how individuals’ hybrid work practices affect teamwork (see also Bell et al., 2023; Raghuram et al., 2019). Hybrid teamwork obviously cannot be treated as interchangeable with conventional co-located teamwork; moreover, it has become increasingly obvious that it also qualitatively differs from our previous understanding of virtual teamwork. Specifically, although it is widely understood that teams vary in their degree of virtuality (i.e., depending on various conditions such as their extent of face-to-face communication), scant attention has been devoted to how the degree of virtuality could vary across *both* team members and time.

However, this is not to say that the vast knowledge that has been built up on team effectiveness and virtual teamwork cannot inform our understanding of hybrid teamwork as it is enacted today. First, there has been a broad discussion on the effects of geographic dispersion between team members (e.g., Foster et al., 2015; Hinds & Mortensen, 2005), including constellations where some members are co-located while others work remotely (O’Leary & Cummings, 2007; O’Leary & Mortensen, 2010). Second, there has been extensive research on the effects of technology dependence as team members coordinate their efforts remotely from different locations (e.g., Dennis et al., 2008; Kirkman & Mathieu, 2005; Maynard et al., 2012). Third, the literature has also acknowledged virtual teamwork experiences go beyond structural factors such as geographic dispersion and technology dependence (e.g., Handke et al., 2021; Watson-Manheim et al., 2012; see also Carlson & Zmud, 1999; Walther, 1992). All of these aspects provide important pieces to the puzzle of hybrid teamwork, yet applications of these theories to hybrid teamwork is still missing. Accordingly, what the field needs in our opinion is a review of how extant knowledge on virtual teams both informs but also limits our understanding of hybrid teamwork. This requires us to clarify what hybrid teamwork actually is, which characteristics are unique to it, and how (or even if) these characteristics are reflected in the current literature.

In this conceptual paper, we seek to advance knowledge on hybrid teamwork in three ways. First, we provide a definition of hybrid teams that involves working out their unique characteristics and distinguishing them from virtual teams. Second, we critically review three broad classes of theories from the virtual teams literature, calling attention to both their contributions and limitations when applied to the context of hybrid teamwork. Third, with these limitations in mind, we map out the most pressing research questions to guide future research on hybrid teamwork.

## Characterizing Hybrid Teamwork

Hybrid work, which refers to any combination of one’s work time spent across organizational and other (typically domestic) work settings (e.g., Grzegorczyk et al., 2021; Hilberath et al., 2020; Sewell & Taskin, 2015), is emerging as a major trend that increases employees’ flexibility in the workplace (e.g., Gibson et al., 2023; Kim & Park, 2023; Sampat et al., 2022). Within the teams literature, hybrid teamwork has typically been vaguely defined as something in between virtual and face-to-face teamwork (e.g., Cousins et al., 2007; Fiol & O’Connor, 2005). Under this view, hybrid teamwork could encompass teams that are co-located (i.e., share the same office space) but only meet up at the office 1 or 2 days a week as well as geographically dispersed teams that schedule in-person meetings a few times a year (Cousins et al., 2007; Mitchell & Brewer, 2022). To further complicate things, hybrid teamwork also extends to teams where some members are co-located while others work remotely, also known as partially distributed teams (e.g., Bos et al., 2009; Burke et al., 1999). For the purpose of this paper, we consider hybrid teamwork as the result of individuals’ hybrid work at the team level, meaning that team members have the possibility of working in a shared office space but can also engage in remote work. As such, we draw on Bell et al.’s (2023) definition of a hybrid team as “*[a team] that regularly switches between having all members co-located and having one or more members working remotely*” (p. 350). However, we note that due to the idiosyncratic nature of individuals’ hybrid work practices it is also possible that team members are never co-located all at once.

Building on this definition, there are two things that uniquely characterize hybrid teams. First, the structural features that have previously been used to describe virtual teamwork—geographic dispersion and technology dependence—are also relevant to hybrid teamwork, yet hybrid teams are also subject to *temporal dynamics*. Through the adoption of different hybrid work practices (i.e., members working from different locations and possibly different times), teams become (more or less) geographically dispersed as a function of individual team members’ remote work. As a result, the modality of intra-team communication (i.e., higher technology dependence, lower degree of face-to-face interaction) will also change as teams coordinate their actions at diverse levels and forms of distance (see e.g., De Guinea et al., 2012; Maznevski & Chudoba, 2000). This may change from week-to-week, day-to-day, or even within-day if an individual spends part of their day at home and part in the office (e.g., to avoid a rush-hour commute or to attend a single meeting in person).

**Figure 1.**
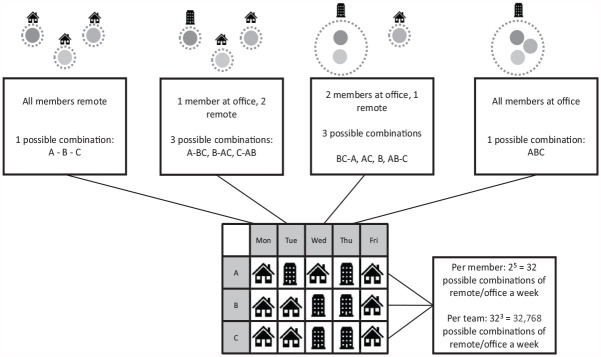
Exemplary illustration of hybrid teamwork combinations in a three-person team over one work week. *Note.* Gray circles symbolize team members A, B, and C. Dashed lines indicate work sites, with several gray circles surrounded by the same dashed line representing co-location at the office.

Second, when and how often individual team members work remotely changes the *configuration* of a team across geographic boundaries. That is, individuals’ hybrid work practices can cause distinct team geographic configurations. For example, we can find situations in which some members form a co-located geographic subgroup at the office, while others are remotely working isolates. Although prior research has discussed the effects of team geographic configuration (see e.g., O’Leary & Cummings, 2007; O’Leary & Mortensen, 2010), the important aspect in hybrid teams is that these configurations can change over time, as team members individually and dynamically switch between working at the office and remotely. For instance, team members A and B may both work at the office on Monday, team member C may join them on Tuesday and all team members may work remotely on Wednesday to Friday—something which could change yet again in the following week. Accordingly, there are both more potentially possible configurations (i.e., there are 2^5^ = 32 possible combinations per individual across a 5-day work week and [2^5^]^team size^ possible combinations per team across the week, see Figure 1)^1^ but also more observed configurations, as these can be in flux from week-to-week, day-to-day, or even within the same day.

In sum, as team members alternate between working at the office and remotely in idiosyncratic week-to-week, day-to-day, or even within-day rhythms, hybrid teamwork is uniquely characterized by both dynamic changes in geographic dispersion (and, as a result, technology dependence) as well as how team members are configured across office and remote locations. In the following section, we will critically review three broad classes of theories within the existing literature on virtual teamwork, reveal how these theories can contribute to our understanding of the two distinguishing characteristics of hybrid teamwork, and discuss their limitations.

## Critical Review

The aim of this critical review is to indicate both how the extant literature on virtual teamwork can be leveraged to understand hybrid teams as well as where it requires expansion, adaptation, or integration to accommodate a clear understanding of hybrid team functioning. We want to represent the virtual work literature as broadly as possible while retaining the detail necessary to describe its relevance to hybrid teamwork. As such, we introduce three broad classes of theories which inform the literature on virtual teamwork. Generally, the literature on virtual teamwork is divided in terms of (a) a (more predominant) focus on structural, that is, more or less fixed and objective features that influence a team’s degree of virtuality, as well as (b) a focus on subjective experiences of virtual teamwork (see Costa & Handke, 2023; Handke et al., 2021). In terms of structural features, the two most frequently adopted and widely accepted dimensions of team virtuality are geographic dispersion and technology dependence (see e.g., Gilson et al., 2015; Raghuram et al., 2019; Schulze & Krumm, 2017). Accordingly, we will review theories on (1) geographic dispersion and (2) technology dependence. Furthermore, we will review theories more grounded in sociomaterial or constructivist traditions and which concentrate on (3) subjective team virtuality experiences.

These broad classes of theories are neither mutually exclusive (for instance, there is an obvious overlap between geographic dispersion and technology dependence) nor is our review an exhaustive account of these or even the virtual teamwork literature more generally. For instance, we recognize other conceptualizations of team virtuality include further dimensions (e.g., functional or cultural diversity, e.g., Chudoba et al., 2005; Ganesh & Gupta, 2010; Gibson & Gibbs, 2006), yet a comprehensive review of the virtual teamwork literature is beyond the scope (and motivation) of the current paper. Instead, we want to give a higher-level consideration of how extant knowledge already accounts for the unique characteristics of hybrid teamwork and where it is still limited (see also Table 1), referring to some seminal publications as representative examples.

**Table 1.** Summary of Contributions and Limitations of the Broad Theory Classes Toward Understanding Hybrid Teams.

	Geographic dispersion	Technology dependence	Subjective virtuality experiences
Contributions	Mapping team geographic configuration at a given time, recognizing formation of subgroups and isolates.	Understanding impact of media choice on team functioning, namely media-task fit. Considering time as relevant for optimal technology usage and its consequences	Relevance of *how* technology is used/appropriated by the team, regardless of its objective features
Limitations	Assumes dispersion is static, and does not account for the temporal dynamics that emerge in hybrid teamwork (i.e., the same team may change remote days over time between team members). Past emphasis on spatial dispersion is less relevant for hybrid teams who are typically located within the same general geographic area and simply alternate remote and in-office work.	Does not account for configural differences in the use of media: - different team members or dyads may have different tech usage. - team members may be using different media simultaneously (e.g., hybrid team meetings) or for the same task (due to some being co-located and others not)	Assumes a compositional model of the construct that requires a shared experience between team members, hence not accounting for a possible configurational model where each team member brings distinct experiences due to the number of office/remote days and to the team members they interact the most as a consequence

### Geographic Dispersion

Within the virtual teams literature, geographic dispersion has been discussed in terms of three underlying dimensions (e.g., Foster et al., 2015; Ganesh & Gupta, 2010; Hill & Bartol, 2016; for an overview, see O’Leary & Cummings, 2007): (1) Spatial (i.e., the average physical distance among team members); (2) temporal (i.e., the extent to which team members have overlapping work hours); and (3) configurational (the way team members are distributed across locations, including the total number of sites the team works from and how many individuals are at each site).

The most notable impact of spatial dispersion is that team members do not (or rarely) share the same physical work environment, which is associated with a range of challenges for team functioning. First, when members do not share the same physical work environment, they engage in less (particularly informal and impromptu) interactions, largely because the effort of interacting with others is higher when there are less spontaneous encounters (e.g., running into each other on the way to the coffee machine). A lack of these interactions, in turn, makes it difficult to share information and establish close and trusting relationships (Fayard & Weeks, 2007; Kraut et al., 1990; Methot et al., 2018, 2021). Second, working in a different physical environment means that members have access to less situational information, which helps them evaluate certain events and behaviors that occur during their work day. Situational information has been shown to contribute to more favorable attributions about one another (e.g., understanding that team member X did not submit their work on time because they were not feeling well and not because they are lazy) and to closer interpersonal bonds (Armstrong & Cole, 2002; Cramton et al., 2007; Hinds & Mortensen, 2005; Kiesler & Cummings, 2002). Third, spatial dispersion means that face-to-face interactions are replaced by technology-mediated interactions. A particular constraint of technology-mediated over face-to-face communication (which we discuss in depth in the section on “Technology Dependence”) is that it transports significantly less (particularly non-verbal) information and thus notably increases the ambiguity of team members’ work environment, making it hard for team members to anticipate each other’s thoughts, feelings, and actions (see Handke et al., 2022). As a result of these challenges, spatial dispersion has been associated with a range of impairments in team processes and emergent states, such as coordination, knowledge sharing, shared mental model development, trust, and conflict (e.g., Hertel et al., 2005; Martins et al., 2004).

These challenges are typically exacerbated through time zone differences and varying work schedules, that is, temporal dispersion. Temporal dispersion significantly affects teams’ ability to engage in synchronous interactions, leading to delays in decision-making and feedback loops, as asynchronous communication becomes the norm (see e.g., Burke et al., 1999; Chudoba et al., 2005). Unlike spatial dispersion, temporal dispersion thus *directly* affects how team coordinate their actions.

Research that has considered geographic dispersion from a configurational perspective has concentrated on how team members are distributed across different work sites. In particular, research in this area has concentrated on the effects of geographically-defined subgroups (i.e., a portion of team members who are co-located at the same site) on overall team functioning, assuming that geographic location is a salient attribute that can trigger social categorization processes (e.g., Fiol & O’Connor, 2005; O’Leary & Mortensen, 2010; Polzer et al., 2006). Specifically, team members may categorize themselves and others based on their physical location (e.g., subgroup A at location X vs. subgroup B at location Y), attributing an in-group status to those who share the same location and an outgroup status to team members at other locations. Through this categorization process, the team’s identity will likely become more fragmented, meaning that team members are more likely to identify with their co-located subgroup than the entire team (see Carton & Cummings, 2012; O’Leary & Mortensen, 2010). These faultlines are problematic in that they reduce the desire of members to exchange knowledge and cooperate with “outgroup” members, resulting in a range of impairments in crucial team processes and states (e.g., conflict, coordination, learning, leadership, Carton & Cummings, 2012; Charlier et al., 2016; Cramton, 2001; Cramton & Hinds, 2004; O’Leary & Mortensen, 2010), as well as performance (Prasad et al., 2017). For instance, O’Leary and Mortensen (2010) found that teams with subgroups had significantly worse team identification, transactive memory, conflict, and coordination than teams without subgroups. These findings were exacerbated if the size of the subgroups was uneven (i.e., imbalanced) across sites.

#### Contribution to Understanding Hybrid Teamwork

When team members work remotely, they need to coordinate their actions across space and possibly also across time. Accordingly, research on the impact of geographic dispersion on team functioning is also informative to hybrid teams when considering how to best coordinate their efforts when working from different locations and possibly also under different work rhythms. However, the most important dimension of geographic dispersion to hybrid teams is undoubtedly configuration, considering that the number of sites in a hybrid team can range between 1 (all members are co-located) to *n* = the number of members in the team (if everyone worked remotely), with numerous possible combinations within this range (see Figure 1). For instance, in a three-person team (Ahmed, Belinda, and Chang), all three could be either co-located or remote, but there could also be any combination of the two of them at the office (Ahmed-Belinda, Belinda-Chang, Ahmed-Chang), as well as any of the three alone at the office. The risk of subgroup formation is thus higher for hybrid than for fully virtual teams, and the number of potential subgroups increases as a function of team size.^2^ Moreover, the number of days that team members spend working remotely also plays a significant role, with the highest risk of geographic subgroups forming at two remote days a week.^3^ Accordingly, knowledge on the nature and effects of geographic dispersion can be informative in understanding the potential for subgroup formation and its associated challenges (e.g., conflict and coordination problems between subgroups).

#### Limitation to Understanding Hybrid Teamwork

Although prior considerations on team geographic configurations are helpful to hybrid teams in terms of considering how patterns of geographic dispersion across different sites could impact team functioning, they are limited in that they do not account for temporal dynamics. Specifically, the inherent assumption has been that team geographic configuration is temporally stable (e.g., team members Ahmed and Belinda always work together at site X while team member Chang works remotely at site Y). However, taking individuals’ hybrid work practices into account means that configurations change over time. Accordingly, configurations that previously distinguished one team from the next could now describe one and the same team but at different points in time. For instance, imagine that team members Ahmed and Belinda work at the same site from Monday to Thursday, Chang joins them on Tuesday and all three work remotely on Friday. In this week alone, the team already exhibits several different geographic configurations—but what if these configurations also changed from week to week?

In sum, the static focus on team members’ work location not only means that we cannot use extant indices of capturing team geographic location (because these are based on temporal stability of individuals’ work sites, e.g., O’Leary & Cummings, 2007) but also that we lack clarity on what actually defines a geographic isolate or subgroup in a hybrid context and which effects these would be associated with. Most notably, prior research has associated team members’ dispersion over different sites with intra-team faultlines and resulting subgroup formation—with detrimental consequences for overall team effectiveness. However, the effects of more dynamic team configurations may be very different. Finally, prior research assumes that isolates have no choice to be isolated and subgroups have no choice to be placed at the same office, and this may be decidedly different for hybrid teams, where individuals may choose to work in co-location or not depending on a range of different factors such as task interdependence, individual constraints (i.e., needing to work from home to attend to family matters or for health reasons), or personal preferences to meet up with some team members but not others.

### Technology Dependence

Most models of virtual team functioning consider technology as an input factor interacting with factors such as task design to shape further team processes, emergent states, and more distal outcomes (e.g., Dulebohn & Hoch, 2017; Martins et al., 2004). Central to the assumed impact of (communication) technology is the degree of informational value (i.e., type and number of cues it is able to transport), synchronicity (i.e., whether information is exchanged in real time or not), as well as the extent to which team members rely on them (e.g., Gibson & Gibbs, 2006; Kirkman & Mathieu, 2005). As Kirkman and Mathieu (2005) argued, while factors such as dispersion are likely to lead teams to communicate electronically, this does not inversely imply that co-located teams will not. That is, while geographically-dispersed teams have to use technology to communicate, co-located teams can also use technology to supplement face-to-face communication (Dixon & Panteli, 2010). For instance, many of us write emails to co-workers working at the same site or even in the same office. We use our smartphone to check emails from work when we are at home, on the train, or even in a meeting. Finally, hybrid work options allow us to work remotely even though we have a perfectly adequate workplace at the office, allowing for many benefits such as scheduling our work more freely, fewer interruptions, and being able to reconcile work with family obligations. Accordingly, considering team virtuality in terms of technology dependence makes it a construct more or less applicable to all organizational teams (see also Gilson et al., 2015; Handke et al., 2018).

Central to the role of technology for virtual team effectiveness is the concept of task-media-fit (e.g., *media richness theory*, Daft et al., 1987; *social presence theory*, Short et al., 1976; *task media fit hypothesis*, McGrath & Hollingshead, 1993). The general idea is that communication effectiveness is determined by how well the informational value of a communication medium fits the informational demands of the task. The informational value of a communication medium is assumed to be determined through its capacity to transmit communication cues both in a high variety as well as at a high velocity. For instance, face-to-face communication enables transmission of not only verbal, but also paraverbal (e.g., intonation) as well as nonverbal (e.g., facial expression, posture) information and is very fast in doing so (in that the sender of a message can obtain direct feedback from its receiver). In contrast, communicating via technology “filters out” some of these cues and/or lowers their transmission velocity, such that some technologies (e.g., emails) can transmit only textual, verbal information at an asynchronous pace. The informational demands of a task, in turn, are generally higher when tasks are more ambiguous or complex and/or when they require a higher degree of interaction between team members. From this perspective, communication media high in informational value (e.g., face-to-face communication) would be more suited to performing ambiguous and interdependent tasks (e.g., negotiating a conflict), while communication media lower in informational value (e.g., emails) would be a fine match for more simple information exchange (e.g., confirming an appointment).

*Media synchronicity theory* (Dennis et al., 2008; Dennis & Valacich, 1999) adopts an even more fine-grained perspective by redefining tasks through their underlying communication processes—convergence and conveyance. Conveyance processes are characterized by transmitting and processing relatively large and diverse sets of information. For instance, a product manager may send out an email to the marketing, sales, and production teams detailing the specifications of a new product, including features, pricing, and launch dates. In contrast, convergence processes are focused on interpretations of pre-processed information to reach a shared understanding with others. Staying with the example above, representatives from marketing, sales, and production may hold a workshop to discuss their strategy, which includes giving feedback on each other’s ideas and planning to ensure all aspects of the project launch are well-coordinated. In other words, consider these processes as “conveyance *of* information and convergence *on* meaning” (Dennis et al., 2008, p. 576). Both processes are more or less required in all tasks, yet in varying degrees and combinations depending on modes of project operation (e.g., inception, execution, see McGrath, 1991) as well as contextual factors such as team familiarity. For instance, experienced teams can already draw on shared goals, roles, and norms from previous projects, so that they require fewer intensive convergence processes that might be more efficient when working through problems and can instead concentrate on conveyance processes while they independently execute their tasks. However, unexpected outcomes may come up over the course of the project that trigger activities necessitating more convergence processes to reach a shared understanding on how to resolve these problems. Communication effectiveness, in turn, is assumed to depend on the fit between need for conveyance/convergence and a communication medium’s synchronicity, that is, the extent which it enables team members to exhibit “a shared pattern of coordinated synchronous behavior,” Dennis et al., 2008, p. 581).

Media synchronicity theory presents a range of different capabilities of communication media that in conjunction determine a medium’s overall synchronicity. These *capabilities* can be regarded as physical properties of a medium and some of them are similar to earlier theories (e.g., transmission velocity, symbol sets, i.e., verbal, paraverbal, or nonverbal language), while others are more adapted to “newer” media (e.g., parallelism, i.e., the extent to which information can be transmitted simultaneously). Media high in synchronicity (e.g., video calls, which show high levels of transmission velocity, and medium levels of symbol sets and parallelism) would be particularly suited for convergence processes, which depend on rapid, iterative transmissions of small quantities of preprocessed information. Conversely, individuals engaging in conveyance processes may need more time to process information (i.e., understand and organize it), meaning that they can (or should) draw on media lower in synchronicity (e.g., digital bulletin boards, which are low in transmission velocity and symbol sets but high in parallelism).

In sum, all theories pertaining to technology dependence convey a common message: communication media differ in their capacity to transport information, whereas tasks (or the processes underlying these tasks) require a certain quantity, quality, and/or frequency of transported information in order to be carried out effectively. Consequently, certain media could be regarded as more suited for certain tasks or communication processes than others. When certain tasks cannot be met with the corresponding media (such as in the case of virtual teams, who cannot meet face-to-face or may have problems organizing video calls because they are located in different time zones), this will impair communication effectiveness. Given that this proposition appears so simple and self-evident, it serves as one of the most widely accepted explanations for (team) virtuality effects.

#### Contribution to Understanding Hybrid Teamwork

The analysis of task-media-fit has greatly contributed to our understanding of technology use and its effects and is thus also highly informative to hybrid teams. Specifically, it can help to understand both team members’ choice to work from a certain work location as well as the effects that this choice can have on team functioning. For instance, when it comes to developing common goals and strategies in new projects, hybrid team members may choose to work at the office rather than remotely in order to more effectively achieve convergence on meaning. Moreover, it can help understand and guide team members’ decisions not only to work remotely but also which media to choose from depending on the task—or more specifically the underlying communication processes—at hand. This allows for a more nuanced perspective on hybrid work than theories on geographic dispersion, which in the case of hybrid teams would differentiate simply between working in co-location or not.

A further contribution by media synchronicity theory in particular is that it acknowledges the impact of temporal dynamics on the degree to which conveyance and convergence will be needed. Specifically, media synchronicity theory suggests not only that communication processes change over the course of a project but that team, task, and media familiarity play a decisive role in the importance of convergence versus conveyance processes. Higher familiarity is generally associated with a lower need for convergence processes, as team members have already established a shared sense of task requirements and activities needed to fulfill these requirements. Media synchronicity theory thus aligns with channel expansion theory (Carlson & Zmud, 1999), which assumes that individuals’ perceptions of communication media change as a function of experience with the medium, task, team members, and organizational context, such that media are perceived as “richer” (i.e., having a higher informational value) when individuals have more experience. In a hybrid team context, this might suggest that teams that are newly formed should plan to be in the office together more frequently, and this can lessen over time. Using other communication channels capable of high synchronicity (e.g., video conferencing) more frequently early on could also fulfill a similar purpose of allowing more time for convergence.

#### Limitation to Understanding Hybrid Teamwork

What is most notable about the theories in this section is that they do not discuss potential intra-team differences in media choice, use, and effects. For instance, although media synchronicity theory speaks of “individuals working with others,” it does not acknowledge that within this group of individuals, there could be differences in convergence and conveyance needs and as a result, of media choice and/or effects. The fact that these theories do not acknowledge teamwork from such a configurational perspective (i.e., recognizing that there could be differences within the team regarding the media team members communicate with) can thus limit our understanding of hybrid teamwork.

Without adopting a configurational perspective, the inherent assumption is that the *entire* team generally (1) engages in the same task or communication processes and (2) uses the same communication media. This assumption is problematic in that it does not account for the various dyadic/subgroup-level interactions within a team, such as those that occur because subsets of team members work together on smaller subtasks, because team members have different preferences in who they interact with, or because of with whom team members are co-located with. The latter is especially important in the case of hybrid teams, who’s members will necessarily have to use different media to communicate with remote versus co-located members. For instance, even when a team is working on the same task (necessitating the same type of communication processes), one might see in-office members discussing potential task requirements face-to-face but using the team’s instant messaging channel for the same purpose with remote team members. Hybrid meetings are another illustrative example, where in-person attendees are collaborating face-to-face with others as well as through the video conferencing platform with the remote attendees. Remote attendees may have varying degrees of inclusion in the meeting and ability to easily speak up throughout. Remote attendees may also be collaborating with one another through the meeting chat, which in-person attendees are unlikely to see or monitor. Accordingly, assuming that some media fit certain task or communication process needs better than others (which can be questioned, see next section), all of the incongruencies in this example are likely to have an effect on overall team communication and thus team functioning.

Moreover, these theories do not consider intra-team differences in team members’ perceived task ambiguity or convergence process needs (which may be the result of individual capacities but notably also of experience in working with one another, the task, and certain communication media) and how this will impact differences in media choice and effects. This is particularly critical in the case of hybrid teams, where hybrid team members may base their work location on their experience. For instance, in a recent study with about 43,000 hybrid workers, Charpignon et al. (2023) reported that newly hired workers were more likely to base their office attendance on their teammates’ or manager’s attendance, suggesting that they may explicitly seek face-to-face communication to compensate for their lack of experience. This is supported by survey results showing that workers spend more of their work time being mentored or mentoring others when they come into the office (Barrero et al., 2021). Accordingly, it seems likely that there are configurational differences in communication media use, which need to be further explored in terms of their effects on team functioning.

### Subjective Team Virtuality Experiences

Overall, both prior classes of theories paint a relatively static and aim for a somewhat “objective” picture of how structural characteristics, such as geographic dispersion or technological features, impact virtual team effectiveness. Even though media synchronicity theory recognizes that individuals can adopt media in different ways (see also adaptive structuration theory, DeSanctis & Poole, 1994), the general assumption behind both the “Geographic Dispersion” and “Technology Dependence” theory classes is that these structural characteristics will have more or less fixed effects on team functioning. This would logically imply that teams who show comparable structural characteristics (i.e., similar degree of dispersion and/or similar technology use) should also be comparable in terms of team processes, states, and outcomes.

However, this is clearly not the case with a range of reviews and meta-analyses showing that the effects of structural team virtuality (i.e., geographic dispersion/technology-mediated communication) are very heterogenous and vary strongly as a function of study setting (most notably laboratory vs. field) as well as team type, tenure, and work design (Carter et al., 2019; De Guinea et al., 2012; Gibbs et al., 2017; Handke et al., 2020; Purvanova & Kenda, 2022). For instance in field studies, where team members work together for a longer period of time (and thus have the opportunity to gain knowledge about each other, the task, and the technologies they work with) and have autonomy in terms of how they coordinate their work, negative effects of structural virtuality are substantially lower (or even disappear entirely) compared to laboratory studies.

Findings of this nature are explained by the last class of theories we discuss here, which are characterized by a social constructivist view that emphasizes *subjective team virtuality experiences* over structural team virtuality. For instance, various of these theories suggest that subjective perceptions of distance are more important than the actual objective separation between team members (e.g., Walther & Bazarova, 2008; Wilson et al., 2008). Accordingly, team members can be geographically dispersed but still feel close to each other and vice versa, with findings showing that perceived—and not physical—proximity is indicative of co-workers’ relationship quality (O’Leary et al., 2014). Moreover, a range of studies suggest that task-media-fit can also dynamically change over time, as team members change the way they use and experience technology (see e.g., channel expansion theory, Carlson & Zmud, 1999; compensatory adaptation theory, Kock, 2001, 2005). For instance, team members may use clearer language to avoid misunderstandings, add emoticons to convey a positive tone, and be less critical of others’ brevity when they know these have a lot of emails to attend to. Accordingly, team members learn to compensate for structural deficits (e.g., using emoticons to compensate for a lack of non-verbal information, drawing on contextual knowledge to explain other team members’ behavior), maintaining high levels of effectiveness without face-to-face interaction.

Examples of contributions within the area of subjective team virtuality experiences include the work of Müller and Antoni (2020, 2021) on shared mental models about information and communication technology (ICT-SMM), or Handke et al.’s (2021, 2024) construct of Team Perceived Virtuality (TPV; for a review, see Costa & Handke, 2023). ICT-SMM refer to a common understanding of how and in which situations information and communication technologies (ICTs) should be used and how they can be appropriated to meet task demands. High levels of ICT-SMM can thus avoid a range of misunderstandings, such as not knowing how often to attend to one’s emails, how to signal the urgency of a specific request, or which technologies are most appropriate to collaborate with on highly interdependent tasks. Avoiding misunderstandings such as these is pivotal to effective team coordination, and ICT-SMM have been linked to higher coordination, performance, and affective team commitment as well as less workload frustration (Müller & Antoni, 2020, 2021).

TPV attempts to integrate structural with social-constructivist elements of team virtuality by concentrating on the experiences that teams make when working together virtually. Building on Watson-Manheim et al.’s discontinuity approach (e.g., Watson-Manheim et al., 2002, 2012), the idea behind TPV is that team members do not experience structural virtuality as problematic per se but only when it disrupts their interactions. Specifically, as soon as members experience disruptions (e.g., communication breakdowns), they will try to make sense of these and their explanations will draw on information from their work environment, which includes—but is not limited to—structural virtuality. This means that TPV is a state associated with subjective team virtuality experiences (in terms of experienced distance and information deficits), rather that fixed, structural properties. Accordingly, TPV *can* occur when team members exhibit high levels of structural virtuality (e.g., because they are geographically dispersed) but it does not have to. For instance, geographically dispersed teams that function smoothly may actually exhibit very low levels of TPV, just as face-to-face teams where members forget to keep each other in the loop about their activities and encounter many frustrating misunderstandings and temporal delays may experience very high levels of TPV. There is a growing body of research related to TPV. For example, Handke et al. (2024) recently found support for TPV’s two-dimensional structure at both individual and team levels of analysis, conceptual and empirical distinctiveness from related team processes and states, and criterion validity regarding both affective and performance-related outcomes. Other empirical work has also found support for constructs that approximate TPV (e.g., Costa et al., 2021, 2024; Gupta et al., 2023), though given the recency of this theory’s development, the literature remains limited at this time.

#### Contribution to Understanding Hybrid Teamwork

Understanding *how* (and not only where) team members work is likely to be important for anticipating and managing dynamics in hybrid teams, as this involves considerations that go beyond the teams’ levels of structural team virtuality. That is, concentrating on actual teamwork experiences also means considering other factors that can be leveraged to improve coordination across multiple sites while still maintaining individual team members’ autonomy in choosing their work location. For instance, hybrid teams should pay particular attention to developing ICT-SMM, to clarify how members at different sites should best communicate, as well as which tasks explicitly require co-location. For example, teams may decide that even when at different sites, they should have virtual check-in meetings in the morning, or that they always meet on site when important strategic decisions need to be made. Reflecting on their perceptions of virtuality can help teams find explanations for communication impairments that do not necessarily relate to their structural virtuality. Handke et al.’s (2021) TPV framework proposes that next to structural virtuality, other factors in the teams’ work environment determine subjective virtuality perceptions. For instance, team familiarity can compensate for high levels of structural virtuality, as team members will have gained sufficient knowledge in how to best interact with one another. At the beginning of their collaboration and when new members join the team, hybrid teams may thus want to introduce periods of higher office attendance, which can then be compensated by higher levels of remote work once sufficient familiarity has been established.

#### Limitation to Understanding Hybrid Teamwork

The work on subjective team virtuality experiences follows a compositional rationale (Kozlowski & Klein, 2000), where these experiences are considered to be *shared* between team members. More specifically, TPV is clearly defined as a team emergent state that is collectively experienced (Handke et al., 2020). In other words, team members’ feelings of distance and information deficits are expected to converge at the team level for a shared perception of virtuality. Similarly, ICT-SMM depend on team members’ common perceptions of technology and thus also suggest that there will be a convergence in perceptions at the team level. However, this conception of sharedness may not necessarily be generalizable to hybrid teams. Most organizational hybrid work policies mandate only a certain amount (rather than fixed days or times) of office attendance (Flex Index, 2024), making it less likely that the entire team is either fully co-located or fully remote. When team members exhibit different office co-attendance patterns, the type and frequency of interactions will also differ between the team members, and consequently, it is very likely that team members will also differ their teamwork experiences. For instance, team members who spend more time together at the office may have more opportunities to gain shared experiences than team members who spend more time working remotely (or who do not encounter as many co-workers on their in-office days), causing differences in perceptual convergence between team subgroups. How this potential configurational perspective influences hybrid team dynamics thus needs to be both conceptually as well as empirically explored.

## Future Research Directions

Grounded in the critical review presented above, we highlight some opportunities for future work on hybrid teamwork. These avenues for future research focus on the two primary characteristics of hybrid teamwork derived earlier in this paper, namely temporal dynamics and configuration. See Table 2 for a summary of key questions to guide future research.

**Table 2.** Questions to Guide Future Research on Hybrid Teams.

Temporal dynamics
• How do continuous changes in individual location and team geographic configurations differentially impact team dynamics at various stages of team collaboration? • How do subgroups and isolates impact team outcomes when team geographic configurations are continuously changing and individuals have some degree of autonomy/choice over work location (i.e., isolates and subgroups may form by choice rather than necessity)? • How do team members adapt their communication as a function of alternating between remote and co-located work? • How can teams best navigate the tension between maximizing individuals’ autonomy in choosing their work location while simultaneously promoting effective coordination at the team level?
Configuration
• What range of potential configurations result from team members’ changes in office attendance patterns, and how can they be quantified in a way that encompasses dynamic changes over time? • How does geographic configuration in hybrid teams impact team communication and collaboration strategies, and specifically how does it impact their use of technology? • Which organization, team, and individual-level antecedents impact hybrid teams’ configurations?

### Temporal Dynamics

More flexible work arrangements may allow individual team members to choose where to work on a daily basis, promoting a continuous change in geographic location on the individual level and a continuous change in geographic configuration at the team level. An important question here is not only how these changes impact team dynamics, but how they may differentially impact team dynamics at different stages of team collaboration. Specifically, hybrid teamwork is likely to differ in both form and effect depending on both the team’s overall stage of development/maturity as well as their phase of task execution (see also Handke et al., 2019; Kirkman & Mathieu, 2005). In terms of team development, co-location is likely to be particularly important during the initial stages in which team members are still getting to know each other and develop a shared identity, or when they encounter conflict or change. Even during later phases of task execution, teams alternate between periods of action and transition (see Marks et al., 2001), and it is during these transition periods, where teams plan tasks or reflect on their taskwork, that teams also require more rapid interaction. Whether this necessarily entails more time in co-location or more fixed hybrid work patterns (where members to know who is at the office and when) still needs to be explored.

Another research avenue is to investigate how subgroups and isolates impact team outcomes when team geographic configurations are dynamic. Past research on team configurations and subgroups and isolates specifically has found negative team outcomes for subgroups and positive ones for isolates (e.g., O’Leary & Mortensen, 2010). However, this research used unchanging configurations in which subgroups and isolates are stable over time, and in which each individual does not exercise choice regularly over where they are working relative to other members of the team. The dynamic nature of hybrid team configurations has several implications for subgroups and isolates that warrant future research. First, there is a need to re-examine exactly what constitutes a subgroup and isolate in hybrid work, because they are likely to be less consistent and stable as team configurations change regularly. Second, any subgroup or isolate that forms is likely at least in part due to individual choice. For example, team members may plan to come in with preferred teammates or avoid other teammates. The implications for team outcomes may differ because of this. For example, while past research has found having an isolate to be a protective factor for team outcomes, in a hybrid team if someone *elects* to work very differently than the rest of the team these findings may not hold and isolates may be associated with more negative outcomes (i.e., if they become socially distanced or ostracized). As such, research should re-evaluate past findings on how subgroups and isolates impact team outcomes when in a hybrid team context.

A further area for future research is to investigate how teams adapt their communication to dynamic changes in work location. Generally, we know that individual-level effects of hybrid work are different than for fully remote work (see e.g., Gajendran & Harrison, 2007; Gajendran et al., 2024; Rudolph & Zacher, 2024) but we still know little about these potential differences at the team level. Yet it is likely that team members will communicate differently when working remotely for only some portion of their work time compared to when they work remotely all of the time. For instance, while fully remote work has been associated with increased feelings of social isolation (e.g., Gajendran et al., 2024; Wang et al., 2021), working remotely only occasionally is often used as a strategy to “get away from them all,” that is, to reduce interactions with co-workers and work with less interruptions (e.g., Thompson et al., 2022; Windeler et al., 2017). Accordingly, team members are likely to adapt their communication as a function of their own, as well as other team members’ office attendance.

Lastly, a pressing question that remains is how teams can best navigate the tension between maximizing individuals’ autonomy in choosing their work location while simultaneously promoting effective coordination at the team level. For instance, when team members are dispersed across various locations and schedules, aligning on tasks, deadlines, and project goals can become challenging. Furthermore, individual autonomy can restrain predictability, making it difficult for team members to anticipate each other’s availability and work patterns. Where can a team then find its equilibrium between the autonomy that individual team members have in choosing where and when to work and the freedom that the team as a whole has in carrying out its tasks (i.e., team autonomy)? In line with prior calls to address the interaction of individual and team autonomy (see e.g., Langfred, 2005; Langfred & Rockmann, 2016), future research should thus focus on how hybrid teams can optimally balance individual flexibility with factors important to team functioning. Addressing this central question could provide insights into designing hybrid work environments that maximize both individual satisfaction and team effectiveness, exploring the conditions under which flexible work arrangements can coexist with the demands of team-based work.

### Configuration

In “traditional” virtual teams, team members’ geographic configuration across space typically does not change. That is, team members more or less either all work from different locations (making them highly similar in terms of dispersion/technology dependence) or are divided up into stable geographic subgroups. In hybrid teams, configurations are much more complex and can range from teams who are completely coordinated in terms of office and remote days (i.e., all members either work together at the office or remotely), while in others team members can switch between being at the office and remote in various ways, such that geographic subgroups not only constantly emerge but also consistently change over time. Future research on hybrid team dynamics thus needs to consider the potential configurations that can result from team members’ (changes in) office attendance patterns. For instance, extant approaches to mapping teams’ geographic configuration, identifying geographic isolates and subgroups, and deriving indices to quantify them (most notably by O’Leary & Cummings, 2007) could be adapted to encompass dynamic changes in configuration. This could include concentrating on the imbalance between specific combinations of co-located team members (rather than between fixed geographic subgroups at different locations, as in extant approaches) and how this relates to subgroup formation. For instance, it seems likely that the risk of subgroup formation in hybrid teams rises when some team members spend more time in co-location than others, yet this relationship remains be explored.

A further area for future research is to investigate how teams’ communication, collaboration, and use of technology varies as a function of team geographic configuration. The way teams communicate and collaborate is likely to differ when all are co-located, all are remote, and when there is a mix of in-office and remote workers. When all team members are co-located, we might expect generally more in-person interaction and somewhat lower dependence on technology. The opposite is likely to be true when all are remote. It is less clear how teams might respond when some team members are distributed and others are co-located. Those who are working together in person may communicate in person and then transmit the message electronically to remote members. Alternatively, they may adopt a “remote first” approach in which communication is done electronically by default unless everyone is in the office together. Future research needs to apply extant knowledge on teams’ technology use to a hybrid team context with special attention to how technology use for communication and collaboration is likely to vary as a function of team configuration.

Lastly, future research could consider which antecedents impact hybrid teams’ configurations, ranging from organizational over team- or even individual-level constraints. At the organizational level, for instance, mandatory office days should play a large role. Out of the 5,859 U.S. companies listed in the Flex Index at the beginning of 2024 (Flex Index, 2024^4^), 21% had a minimum office day requirement. Out of these companies, in turn, 51% mandated that employees come into the office at least 3 days a week, which statistically increases the number of possible configurations. Conversely, mandating that employees come into the office on specific days (so-called “anchor days”; employed by only 9% of listed companies) necessarily decreases the number of possible configurations. At the team level, task interdependence has been identified as a crucial boundary condition for virtual team effectiveness (e.g., Handke et al., 2020, 2021; Kanse et al., 2023) as well as for the impact of both individual and team autonomy on team performance (Langfred, 2000, 2005). High task interdependence typically requires more interaction between team members to complete their task, and this interaction is usually more effortful in distributed compared to co-located settings. Accordingly, hybrid teams with high levels of interdependence may try to maximize office co-attendance, at least between those members of the team who need to work together particularly closely for a given task. Finally, individual-level constraints can also impact hybrid teams’ configuration. For instance, members with higher preferences for segmentation (i.e., preference to keep aspects of work and home domains separate from one another, Ashforth et al., 2000; Kreiner, 2006) or socializing with co-workers will spend more time working at the office, while those with care responsibilities will often have to work from home—even if they consider it to be less fruitful for the team or to them personally. Moreover, friendship/liking between certain team members could also be a decisive factor, as individuals will be more likely to go to the office if people they like are also there—or avoid coming into the office if they are not.

## Notes

1. Assuming that there are two working modes per individual (i.e., office vs. remote) and 5 days a week on which individuals can choose whether to work remotely or at the office, there are 2^5^ different combinations of hybrid work modes across the week per individual. Raised to the power of the number of team members, this then corresponds to the number of possible combinations per team across a 5-day work week. For instance, in a team of three members, there are 32^3^ = 32,768 possible configurations for a given 5-day work week with team members working either remotely or at the office per day—ranging from all team members working remotely over different combinations of remote and office work to all team members being co-located at the office (see Figure 1).

2. The number of potential subgroups within a team can be calculated with the formula *n*!/(*k*! × (*n*−*k*)! (with “*n*” being the total number of team members and “*k*” being the number of members in subgroup. For instance, in a team of three members, a subgroup would consist of two members. Applying the formula above gives us 5!/(2! × (5−2)! = 3 different subgroup combinations. In a team of five members, there could be a subgroup of two, three, or four members, amounting to [5!/(2! × (5−2)!] + [5!/(3! × (5−3)!] + [5!/(4! × (5−4)!] = 10 + 10 + 5 = 25 different possible subgroups.

3. The likelihood of a team member being at the office on a given day of the 5-day work week is (5−*r*)/5 and the likelihood of them working remotely is *r*/5 (with “*r*” being the number of remote work days). In a three-person team, the likelihood of two members forming a co-located subgroup at the office would then be ((5−*r*)/5) × ((5−*r*)/5) × (*r*/5), multiplied by the number of different possible combinations (in a team of three members: three). Accordingly, for one remote work day a week, the likelihood of a co-located subgroup forming is 3 × [(4/5) × (4/5) × (1/5)] = 0.38, while for two remote work days a week, the likelihood of a co-located subgroup forming is 3 × [(3/5) × (3/5) × (2/5)] = 0.43.

4. These examples are drawn from the Flex Report and depict the status quo in Q1 2024. They can thus be subject to change. The current version of the Flex Report can be accessed here: https://www.flex.scoopforwork.com/
